# Implementation of a lung cancer screening program at an urban safety-net hospital: enabling solutions via stakeholder engagement

**DOI:** 10.3389/fpubh.2026.1751534

**Published:** 2026-08-06

**Authors:** Stephen J. Kuperberg, Jonna Mercado

**Affiliations:** 1 Division of Pulmonary and Critical Care Medicine, Department of Medicine, New York City Health and Hospitals, Woodhull, Brooklyn, NY, United States; 2 Department of Medicine, New York University Grossman School of Medicine, New York, NY, United States

**Keywords:** barriers, disadvantage, implementation, lung cancer, prevention, safety-net, socioeconomic, vulnerable

## Abstract

Lung cancer screening (LCS) has been shown to reduce lung cancer mortality; however, numerous obstacles impede the successful development and expansion of LCS programs, particularly in underserved communities where uptake is limited. Neighborhood disadvantage plays a significant role, as numerous studies have elucidated the influence of race, ethnicity, income, and other measures of social disadvantage on LCS, as well as lung nodule surveillance programs. Successful development and expansion of LCS and nodule programs in inner-city hospitals can be an important means of improving health equity. The present commentary describes the key elements, challenges, and solutions involved in the successful implementation of an LCS program in an urban safety-net hospital, focusing on high-impact relationships with intra-institutional and system-level stakeholders.

## 1 Introduction

Lung cancer screening (LCS) holds great promise for mortality reduction, as evidenced by decades of robust data from numerous trials, including the National Lung Screening Trial (1), the Nederlands-Leuvens Longkanker Screenings Onderzoek (NELSON) trial (2), and DANTE trial (3). The United States Preventive Services Task Force adopted these findings in its recommendations in 2013 (4). Eligibility expansion enabled greater reach for screening benefits because the modified criteria (5, 6) widened the number of eligible participants by broadening the age range from 55–74 to 50–80 years and reducing the pack-year requirement (7). Despite this trend, disparities exist in nodule evaluation programs and LCS efforts across ethnic and socioeconomic groups (8, 9). This highlights the pressing need for improved implementation of such programs for high-risk patients, especially in underserved areas where limitations in health care access are evident (8, 10). Among the most influential social determinants of health driving health care inequity in lung cancer evaluation are race and ethnicity. Numerous studies have shown that individuals who identify as Black are diagnosed with lung cancer at a later stage and are less likely to be offered early surgery after diagnosis (8, 11–13). Other key social determinants of health, such as geography (14) and income (15) are correlated with magnified disparities in LCS uptake, impacting lung cancer incidence, and mortality (16). Unfortunately, variability in LCS uptake and follow-up exists, with starkly uneven distribution in access across neighborhoods and regions (17, 18). Thus, combating access disparities involves improvement and expansion of LCS and incidental lung nodule (ILN) programs, especially in medically underserved neighborhoods. Despite significant resource constraints, these numerous hurdles were successfully overcome throughout our implementation process, ultimately resulting in a sustainable model that may serve as a blueprint for successful LCS program development in similar healthcare settings. Thus, as a safety-net hospital serving vulnerable populations that are disproportionately affected by barriers to healthcare access, we believe that it will provide value from a public health standpoint to inform the literature regarding our unique experience in LCS program development, including community context, description of successful strategic methods, logistical challenges, and accompanying solutions.

## 2 Context

Within the implementation landscape, numerous obstacles exist that may hamper LCS program development and expansion. Woodhull Hospital is a community academic hospital in a densely populated area in Brooklyn, New York. It is one of 11 full-service hospitals within the New York City Health and Hospitals (NYCHH) system, the largest municipal hospital network in the United States, consisting of over 45 sites (19). Woodhull is situated in a medically underserved neighborhood at the intersection of the Bedford-Stuyvesant and Bushwick neighborhoods, a designated health care provider shortage area (20). Its zip code, 11,206, alone has a population density of over 61,000 person per square mile (21). At the state level, New York has a poverty rate higher than the national average (22). Notably, in 2021, the poverty rate in Bedford-Stuyvesant was 24%, whereas that in New York City was 21%.

At the time of the program's initiation in 2021, our institution lacked a formal Pulmonary Medicine division, nor possessed an LCS program, largely owing to resource gaps amplified by provider shortages following the COVID pandemic. With the formation of a new pulmonary faculty cohort with Interventional Pulmonology training and LCS program development experience (SK), and considering the robust evidence that LCS reduces lung cancer mortality, the development of a formal LCS program by the authors (SK, JM) was deemed logical and necessary.

## 3 Programmatic elements

As with all LCS programs, challenges exist in implementation at the institutional, provider, and patient levels (Table 1). The first challenge in developing an LCS program at Woodhull was organizational, requiring support from both local hospital leadership and the parent system, NYCHH (9). At both the parent-system and local levels, evidence from the literature highlighted the need for the implementation of a local LCS program (8, 23, 24). A focus emerging from discussions was the lack of an LCS program in the hospital, representing a key gap in oncologic care. At the time of initiation, LCS programs in New York City had been widely adopted by private systems (25), but their rollout in the public sector was generally limited. This limitation reflected a nation-wide deficit in 2022, whereby only 5.8% of eligible persons were reported to have been screened (26), underscoring the need for program creation. For these reasons, coupled with the recent recruitment of pulmonary medicine faculty with expertise in interventional pulmonology and onco-pulmonology (27) collaborative efforts were undertaken to engage hospital stakeholders, including leadership, administration, finance, radiology, pulmonary medicine, primary care, information technology, and ancillary services. This initial engagement led to the assembly of a core team to launch the LCS program. The team conducted an in-depth review of national guidelines and requirements for LCS programs (28, 29), covering quality measures such as reporting, data collection, privacy, and security. Logistics of the screening process were discussed across multiple meetings, with attention to diagnostic avenues for biopsy of high-risk lesions, including interventional pulmonology, interventional radiology, and thoracic surgery.

**Table 1:** Challenges and solutions encountered during LCS program development.

Challenges	Next step	Solution/action plans
Technology utilization	Needs assessment	Migrate from DynaLync to EPIC
	Workflow/schematic	Participate in system-wide discussion of advanced use of EPIC for tracking and prevention of loss to follow up.
Staffing	Stakeholder meeting	Hiring of mid-level provider
	Provider time analysis	
Resources allocation	Business plan: meeting with senior staff/financial officer, system leadership.	Resource allocation by leadership, hiring
Network parity	Council meetings	Communication with central/system leadership
Outreach	Site visits, educational programs in network and community	Face-to-face and tele-video office visits with neighborhood clinics
		Patient/provider education during the community-based meetings
Provider education	Grand rounds, email communication	Hospital-wide grand rounds, system council meetings, information dissemination
Components: Smoking cessation program, shared decision-making	Program development: smoking cessation	PCP education on smoking cessation
	Assess collaborative role with primary care providers (PCPs)	EPIC optimization for smoking cessation referral
		Enrollment of mid-level providers in smoking cessation courses/clinical champions for smoking cessation.
	Assess collaborative role of mid-level providers (e.g., nurse practitioners and physician assistants) for evaluation and treatment	Administrative support for template management for scheduling

PCP, primary care provider.

During program development, a workflow of diagnostic and treatment pathways was outlined based on the Lung Imaging and Reporting Data System LUNG-RADS (30) determination defined by the American College of Radiology. A multidisciplinary tumor board review consisting of referral end points, including thoracic surgery, oncology, and radiation oncology, were formalized, and a written protocol was designed to highlight the position of each referral service.

As part of the feasibility and auditing phase, careful review of prior internal program capabilities revealed that the existing mammography screening program was a reference point for the LCS workflow. Specifically, the mammography workflow within the radiology department revealed a structure consisting of staffing and radiology-specific elements such as a concrete workflow for positive findings. Careful evaluation of the aforementioned components and their adaptation to align with health-system requirements resulted in the development of a preliminary framework for establishing a parallel LCS program.

Infrastructure assessment then followed, covering hardware, computed tomography (CT) scanner volume capability, expected number of scans, and software compatibility (e.g., the network-based tracking database DynaLync (Philips, US). Based on the outcomes of these meetings, the team formulated strategies for financial and human resource allocation, recruitment, and workforce training to ensure sufficient staffing capacity for program implementation. A vital element of program development was ensuring the presence of sufficient personnel to process the influx of new scans and referrals to enable clinical follow-up and avoid delays in care. The technical expertise of the interpreting radiologist was confirmed to ensure that the LCS readings fully conformed to the recently updated LUNG-RADS interpretation system (31).

Finally, a hospital-based schematic for the program's workflow was required, which accounted for patient time and volume, and scrutinized the system to identify resource gaps that needed to be addressed to accelerate the program. This schematic outlined the roles of key stakeholders and the sequence of clinical processes, beginning with provider-initiated low-dose CT (LDCT) scan ordering and progressing through risk stratification, multidisciplinary tumor board review, and appropriate follow-up management, including surgical referral, diagnostic procedures, or repeat imaging (32).

Beyond structural needs, funding and administrative support were additional obstacles to the launch of the LCS program. To overcome these challenges, a human and technical needs assessment was incorporated into an LCS program proposal and submitted to hospital leadership. Subsequently, pre-launch meetings accompanied by frequent inter- and intra-departmental communication were employed to identify existing personnel with skillsets relevant to the technological components of LCS. Once an internal workflow was identified and agreed upon, a process that defined two-way communication of LUNG-RADS data with the parent system was developed to ensure that LCS data were stored centrally, locally, and securely in line with the Health Insurance Portability and Accountability Act.

Patient tracking was performed under the purview of an LCS navigator, an essential component of the program. LCS navigation has been widely demonstrated to be an evidence-based means of improving uptake, especially in underserved areas; accordingly, it was established as a central component of program development (33, 34). At the time of program launch, internal funding to hire a navigator was unavailable; therefore, a review of personnel resources identified a highly motivated clinic staff member with expertise in care coordination and community outreach to adopt the role following expedited training. This adaptation was pivotal to program development, serving as a catalyst for program growth while enhancing patient data tracking and communication. Although not a major obstacle to program development, current procedural terminology and International Classification of Diseases codes, along with their payment pathways, required delineation and reconciliation with accurate billing practices to ensure coverage and payment. These efforts were particularly important for facilitating access to care and minimizing the risk of financial burden among our frequently underinsured patient population.

Once feasibility was established via communication with our parent system (NYCHH), administrative support was granted in the form of a formally trained LCS nurse practitioner (LCSNP) to assist with clinical and administrative aspects of the program. The addition of the LCSNP was invaluable, leading to improved organization of LCS data, enhancements in patient care, reduced wait times, and a clear bolstering of team dynamics and morale. Based on established findings (34), the deployment of a patient navigator emerged as a critical lever of operational improvement. Although no patients were enrolled prior to program establishment, we planned to quantify success based on multiple key variables. The absolute value of enrollees was tracked on a quarterly and annual basis. The total number of scans obtained was simultaneously measured, and the completion rate (defined by number of scans completed/number of scans ordered × 100%) was measured using the “Slicer Dicer” feature of the electronic medical record. Notably, incongruence of process variable tracking between internal program data collection and system-wide software implementation was a key challenge. This was ameliorated by using accessible variables such as total enrollees and completion rate (Table 2).

**Table 2:** Outcome metrics during the first 3 years of the program.

Metric at year 3	Value	Calculation method
Total enrollees in LCS at Q4 of year 3	1362 patients	EPIC slicer dicer
Total LDCT scans completed	2035 LDCT scans	EPIC slicer dicer
Completion rate (scan ordered/scan completed)	68.2%	EPIC slicer dicer
Cancers diagnosed	52	Internal data collection/pathology dept

Source, EPIC slicer dicer; LCS, lung cancer screening; LDCT, low-dose computed tomography.

A final crucial step in the LCS launch was engagement of primary care providers within the institution and surrounding community. This was essential because of the lack of pre-existing LCS or lung nodule programs in our facility and the resulting absence of referral patterns. Given that primary care providers (PCPs) encounter the majority of individuals eligible for LCS, educational programs focused on LCS workflow are imperative to reinforce knowledge, emphasize screening mandates such as shared decision-making, and clarify referral streams (24). Collectively, these efforts contribute to overcoming healthcare disparities. Therefore, seamless integration of PCPs into the LCS program was a priority. A grand rounds lecture was a particularly effective strategy for provider engagement and education. In addition, program staff disseminated educational materials in PowerPoint format and distributed targeted email communications to reinforce key program components and workflow processes. Finally, word-of-mouth discussions and one-to-one teaching sessions were provided to disseminate program knowledge. Through these communication modalities, we fully conveyed aspects of the program, including referral workflows and patient selection. Ultimately, these efforts substantially increased program awareness and visibility and were instrumental in facilitating successful program launch.

Now following its complete implementation, the Woodhull LCS program has organized tracking, reporting, and clinical components both internally and within the parent system. A clinical research component is integral, which involved performing an observational study at the time of the program inception. For example while epidemiologic information pertaining to LCS *per se* was still being aggregated in the first 2 years, we retrospectively investigated patients with suspicious lung nodules from 2020 to 2022 (i.e., prior to LCS program initiation) who also had a high prevalence of tobacco use (74%) and chronic obstructive pulmonary disease (34%). We found that the most influential socioeconomic factors in this cohort were country of origin and insurance status, and that non-commercial insurance status was associated with larger lung nodule size at presentation.

Today, the Woodhull LCS program remains integrated with the hospital's nodule clinic and bronchoscopy team, enabling comprehensive lung cancer evaluation through nodule assessment, multidisciplinary tumor board review, and a well-defined referral pathway to cardiothoracic surgery for patients requiring potential surgical resection. Robotic bronchoscopy (35) and cryobiopsy (36), both essential tools for lung cancer diagnosis, have been added to the program's armamentarium of diagnostic modalities, bolstering its ability to rapidly diagnose lung cancer or effectively rule it out when necessary.

## 4 Discussion and future directions

Despite numerous challenges, successful program implementation was achieved through sustained collaboration among LCS stakeholders and evaluation of local capabilities and technical expertise. Together, these activities generated creative and comprehensive solutions that enabled program implementation. Since its inception, the program has evolved in terms of staffing, resources, and workflow optimization, enabling over 1,300 patients annually to access LCS and preventive care services at our institution alone. The importance of these efforts is underscored by persistently low uptake of LCS among eligible individuals—for example, only 17% in a recent large cohort (37)—as well as by even lower participation rates among marginalized populations (18, 23, 37, 38), forming the basis for expansion of LCS in the underserved community surrounding Woodhull Hospital.

Achieving the mortality reduction benefits of LCS in urban healthcare settings requires the development of well-functioning “*de novo*” programs that effectively address disparities in LCS associated with socioeconomic deprivation. A chronologically based summary of the processes involved is presented in Table 1. Notably, clear and detailed communication with our parent system increased program visibility and funding, resulting in staff supplementation with a dedicated LCS navigator, which exponentially expanded the program's reach and ability to engage stakeholders. The result was measurable improvements in key performance indicators, including overall number of enrollees and LDCT completion rates (Table 2). Program elements, including workflow optimization strategies, were subsequently adapted to inform the development of similar programs at sister facilities across the health system.

Key limitations in our experience included provider shortages leading to severe time constraints, lack of support staff and mid-level providers, and limited community engagement in the planning phase. Greater incorporation of community input during program development may have further strengthened workflow design and operational effectiveness. Furthermore, as the COVID-19 pandemic persisted into 2021, competing demands related to intensive care unit staffing diverted resources and staff. Nevertheless, ongoing patient engagement and feedback provided valuable insights that informed iterative improvements and ultimately contributed to the program's continued refinement and success.

To enhance generalizability, modifiable workflow components, including navigator staffing levels, communication strategies involving both on-site and satellite physicians, and integration of trainees and mid-level providers, can be adapted and scaled to meet the needs of larger healthcare systems. Notably, comprehensive education of staff and patients, coupled with implementation of successful tracking mechanisms, are imperative for patient retention and minimizing loss to follow-up. Effective tracking mechanisms may also reduce the risk of missed lung cancer diagnoses and, consequently, influence mortality outcomes (39). Furthermore, technological adaptability, optimization of electronic health records, and integration of artificial intelligence–based methods (40–42) may serve as important facilitators throughout the continuum of LCS program development (43). Indeed, an imperative within new and existing LCS programs in underserved communities is the need to move toward precision methods to include and personalize the care for patients who may benefit from screening (44). Such methods include software analysis of imaging features suggestive of malignancy (i.e., “radiomics”) and serologic sampling for blood-based biomarkers, which can bolster the capability of growing programs to incorporate screening models which reduce the rate of over- or under -diagnosis of malignant nodules detected. Precision medicine can thereby assist programs population-specific screening inconsistencies such as overdiagnosis of ground glass nodules in Asian cohorts (45) and under-screening of Black individuals who face screening disparities due to lower reported tobacco “pack-years” (46).

## 5 Conclusions

The development of well-designed LCS programs in medically underserved areas is essential to addressing disparities in screening access. Developing novel, highly functional LCS programs may provide a clear pathway to better patient-specific survival, improved health equity, and improved population health through reduced lung cancer mortality (8, 10, 47, 48). We propose that success derived from the ideological desire to provide LCS services, coupled with iterative efforts in team-building, internal process review, and persistent engagement of intra-institutional and system-wide stakeholders from interconnected disciplines (e.g., hospital administration, central Office administration, information technology, radiology, oncology, thoracic surgery).

Functional operations required study of untapped resources within the hospital; and prioritization of community, patient, and provider education. Our experience in LCS program implementation revealed numerous logistical challenges, however the solutions utilized to overcome them described here may be valuable if taken together and reproduced for programmatic success. Key elements included frequent engagement of stakeholders for education dissemination, process improvement, quality checks, quantitation of metrics, and clinical education. Finally, LCS navigator inclusion and frequent community engagement events were pivotal for optimal program growth.

Although the LCS program was developed within a centralized healthcare system serving an underserved inner-city hospital, our model can be adopted and applied to other underserved populations experiencing regional disparities, particularly in rural settings (49). High-risk patients in rural regions face distinct barriers to both LCS and follow-up care (17, 50), including higher smoking prevalence and greater geographic distance from screening facilities. Consequently, rural communities represent an important target for systematic program improvement (51), where stakeholder engagement and tailored implementation strategies are critical in improving access and outcomes.

Further research on LCS program implementation in resource-constrained locations should include qualitative assessments of patient perspectives and experiences, interface with local diagnostic programs such as bronchoscopy, and the role of multi-disciplinary meetings. These evaluations should ensure distinction of outcomes across socioeconomic strata.

Operationally, given the emerging understanding of disparity between centralized and de-centralized LCS programs (43, 52, 53), qualitative assessments of stakeholder roles during program implementation in each setting may provide valuable insights into factors associated with program success. Such findings could inform the design and implementation of future programs and be evaluated against quantitative performance metrics, including screening enrollment and adherence to the spectrum of guideline-based recommendations (54, 55).

**Figure 1:**
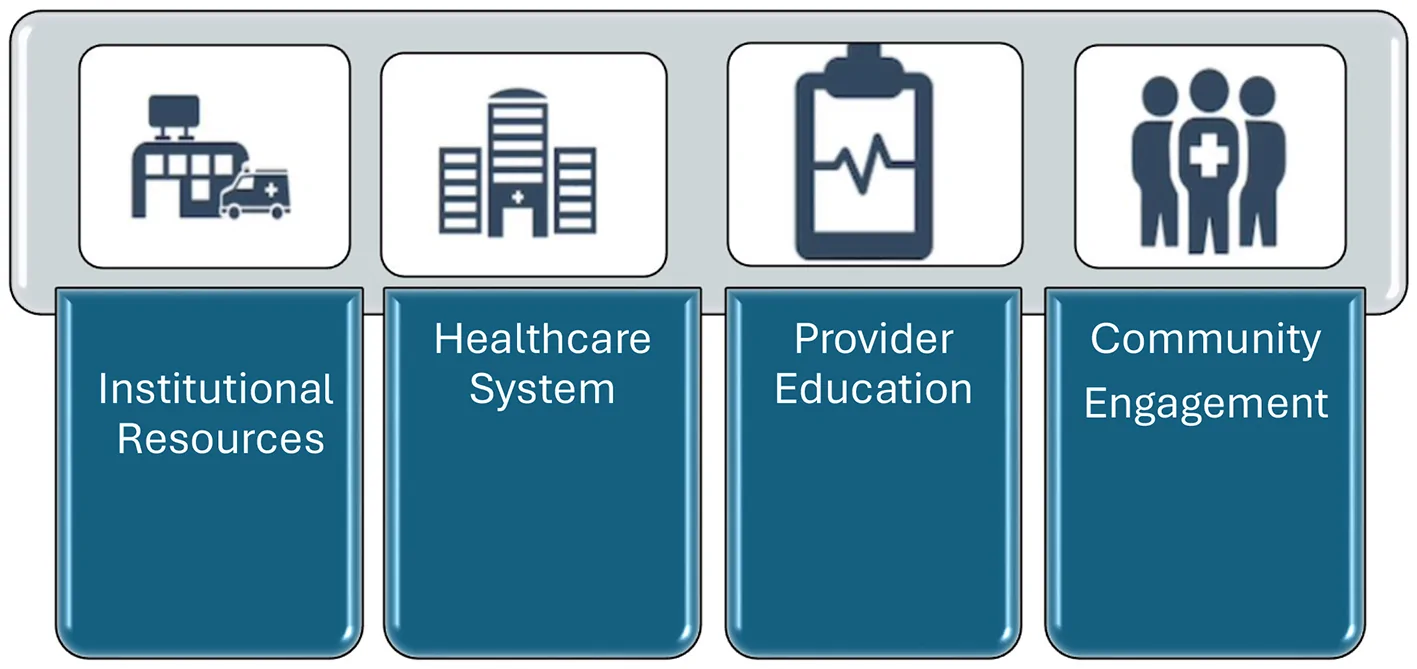
Pillars of LCS program development in an urban safety-net hospital.

Finally, objective evidence for the effectiveness of the aforementioned strategies is seen in the large number of enrollees in our LCS progress during the first 3 years and notable completion rate, as listed in Table 2. The lessons learned from our experience can be a basis for establishing programs “*de-novo*” in under-resourced areas and improving existing programs (Figure 1). Ultimately, we propose that stakeholder-driven solutions are vital for establishing and sustaining effective LCS programs in vulnerable communities.

## Data Availability

The original contributions presented in the study are included in the article/supplementary material, further inquiries can be directed to the corresponding author.
